# Spatial patterns and secular trends in human leishmaniasis incidence in Morocco between 2003 and 2013

**DOI:** 10.1186/s40249-016-0135-8

**Published:** 2016-05-11

**Authors:** Mina Sadeq

**Affiliations:** Environmental Epidemiology Unit, National Institute of Hygiene, Ministry of Health, 27 Avenue Ibn Battuta, BP 769 Rabat, Morocco

**Keywords:** Human leishmaniasis, Epidemiology, Incidence rates, Spatial patterns, Secular trends, Morocco

## Abstract

**Background:**

Few studies on spatial patterns or secular trends in human leishmanias have been conducted in Morocco. This study aimed to examine spatial patterns and trends associated with the human leishmaniasis incidence rate (HLIR) at the province/prefecture level between 2003 and 2013 in Morocco.

**Methods:**

Only the available published country data on the HLIR between 2003 and 2013, from the open access files of the Ministry of Health, were used. Secular trends were examined using Kendall’s rank correlation. An exploratory spatial data analysis was also conducted to examine the spatial autocorrelation (Global Moran’s I and local indicator of spatial association [LISA]), and spatial diffusion at the province/prefecture level. The influence of various covariates (poverty rate, vulnerability rate, population density, and urbanization) on the HLIR was tested via spatial regression (ordinary least squares regression).

**Results:**

At the country level, no secular variation was observed. Poisson annual incidence rate estimates were 13 per 100 000 population (95 % *CI* = 12.9–13.1) for cutaneous leishmaniasis (CL) and 0.4 per 100 000 population (95 % *CI* = 0.4–0.5) for visceral leishmaniasis (VL). The available data on HLIR were based on combined CL and VL cases, however, as the CL cases totally outnumbered the VL ones, HLIR may be considered as CL incidence rate. At the provincial level, a secular increase in the incidence rate was observed in Al Hoceima (*P* = 0.008), Taounate (*P* = 0.04), Larache (*P* = 0.002), Tétouan (*P* = 0.0003), Khenifra (*P* = 0.008), Meknes (*P* = 0.03), and El Kelaa (*P* = 0.0007), whereas a secular decrease was observed only in the Chichaoua province (*P* = 0.006). Even though increased or decreased rate was evident in these provinces, none of them showed clustering of leishmaniasis incidence. Significant spatial clusters of high leishmaniasis incidence were located in the northeastern part of Morocco, while spatial clusters of low leishmaniasis incidence were seen in some northwestern and southern parts of Morocco; there was spatial randomness in the remaining parts of the country. Significant clustering was seen from 2005 to 2013, during which time the Errachidia province was a permanent ‘hot spot’. Global Moran’s I increased from 0.2844 (*P* = 0.006) in 2005 to 0.5886 (*P* = 0.001) in 2011, and decreased to 0.2491 (*P* = 0.004) in 2013. It was found that only poverty had an effect on the HLIR (*P* = 0.0003), contributing only 23 % to this (Adjusted R-squared = 0.226).

**Conclusion:**

Localities showing either secular increase in human leishmaniasis or significant clustering have been identified, which may guide decision-making as to where to appropriately allocate funding and implement control measures. Researchers are also urged to undertake further studies focusing on these localities.

**Electronic supplementary material:**

The online version of this article (doi:10.1186/s40249-016-0135-8) contains supplementary material, which is available to authorized users.

## Multilingual abstract

Please see Additional file 1 for translations of the abstract into the six official working languages of the United Nations.

## Background

Leishmaniasis is a vector borne disease, which has a wide clinical and epidemiological diversity with respect to leishmania parasite types, vector species, and reservoirs. In Morocco, leishmaniasis is a public health concern [1]. Parasitological information on leishmaniasis in Morocco is documented (see Table 1). Notification of leishmaniasis is mandatory (according to the act *Arrêté ministériel N° 683–95 (31 mars 1995)*), a leishmaniasis control program (both for cutaneous [CL] and visceral leishmaniasis [VL] was established in 1997 [2], and there are also vector control and reservoir control programs in place [3]. From 1997 to 2000, diagnosis laboratories were established, which improved surveillance and diagnosis of cases since 2001, particularly for CL [4]. Between 1997 and 2013, the mean annual estimate of reported cases was 3 028 per year for CL, with a maximum number of 8 707 cases reported in 2010 and a minimum number of 571 cases reported in 1999. For VL, the mean annual estimate of reported cases was 121 cases per year, with a maximum number of 170 cases reported in 2006 and a minimum number of 69 cases reported in 1998 [4]. Poisson annual incidence rate estimates for the period of 2003–2013, calculated as part of this study, were 13 per 100 000 population (95 % *CI* =12.9–13.1) for CL and 0.4 per 100 000 population (95 % *CI* = 0.4–0.5) for VL. It is well known that CL cases significantly outnumber VL cases [4, 5], the annual mean number of which during 2003–2013 was 135 (107–170) cases, with a standard deviation of 24. This might be the reason why leishmaniasis-related data published by the Service of Studies in Heath and Health Information (SSHHI)-Ministry of Health [4], at the provincial level, include the total number of leishmaniasis cases, combining CL and VL cases. One of the ambitious challenges of the Moroccan Ministry of Health, part of its 2013–2016 strategy, is “to halve the incidence of *Leishmania tropica,* and to interrupt transmission of cutaneous leishmaniasis due to *L. major* in concerned foci, by the end of 2016” [7].

**Table 1:** Parasitological information related to leishmaniasis in Morocco

Leishmania species	Clinical form	Vector species	Reservoirss
*L. major*	*ZCL*	*P. papatasi*	*Meriones shawi, Psammomys obesus*
*L. tropica*	*CL*	*P. sergenti, P. chabaudi*	*Human Canis familiaris*
*L. infantum*	*ZVL, CL*	*P. perniciosus*	*Canis familiaris P. ariasi, P. longicuspis*

**Source:** Dr Laamrani El Idrissi Abderrahmane, Ministry of Health-Morocco. WHO Consultative meeting on Cutaneous Leishmaniasis in EMRO countries, Geneva, 30 April to 2 May 2007 [3]

**Fig. 1:**
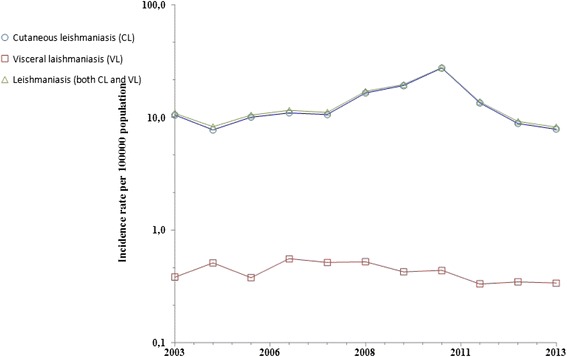
Incidence rate (Log10 scale) of human leishmaniasis, Morocco, 2003–2013. This figure depicts secular trends of human leishmaniasis at the country level from 2003 to 2013. There is a perfect overlap between HLIRs (CL and VL combined) and CL incidence rates

The incidence of human leishmaniasis varies over time and place due to many factors, including urbanization, social factors such as poverty, and others [8], or as a consequence of the poor implementation or evaluation of control strategies. In Morocco, the annual incidence rates, reported by the SSHHI-Ministry of Health, showed no statistically significant increasing or decreasing trends covering the period from 2003 to 2013 for either CL or VL (*P*-values > 0.05) (see Fig. 1). Leishmaniasis is endemic in many Moroccan localities, but in recent years, cases have also emerged in new areas. Very few studies [6] showing geographical distribution of human leishmaniasis have been carried out in Morocco [3, 6]. No spatial analysis techniques to analyze leishmaniasis incidence, such as spatial clustering/autocorrelation, have been used in the country as yet, and leishmaniasis-related secular time trends at the province/prefecture level have also not yet been explored. The overall national strategy to deal with leishmaniasis lacks powerful scientific tools/approaches such as GIS and spatial analysis techniques [7].

To update and better understand leishmaniasis epidemiology in Morocco, in order to consequently guide decision-making in terms of where to allocate funding and target prevention and control efforts, a spatial analysis of the disease to identify spatial clusters and how they change over time is required. This study was aimed at examining spatial patterns and trends in human leishmaniasis at the province/prefecture level, covering the period of 2003–2013, in Morocco, including in the south of the country for which leishmaniasis-related published information has been scarce. Associations between the human leishmaniasis incidence rate (HLIR) and 1) urbanization, considering ‘province’ and ‘prefecture’ as the two degrees of urbanization; 2) population density; and 3) social factors, including ‘poverty rate’ and ‘vulnerability rate’ as covariates, were examined.

## Methods

### Study area

#### Features of the study area potentially relating to leishmaniasis incidence

**Fig. 2:**
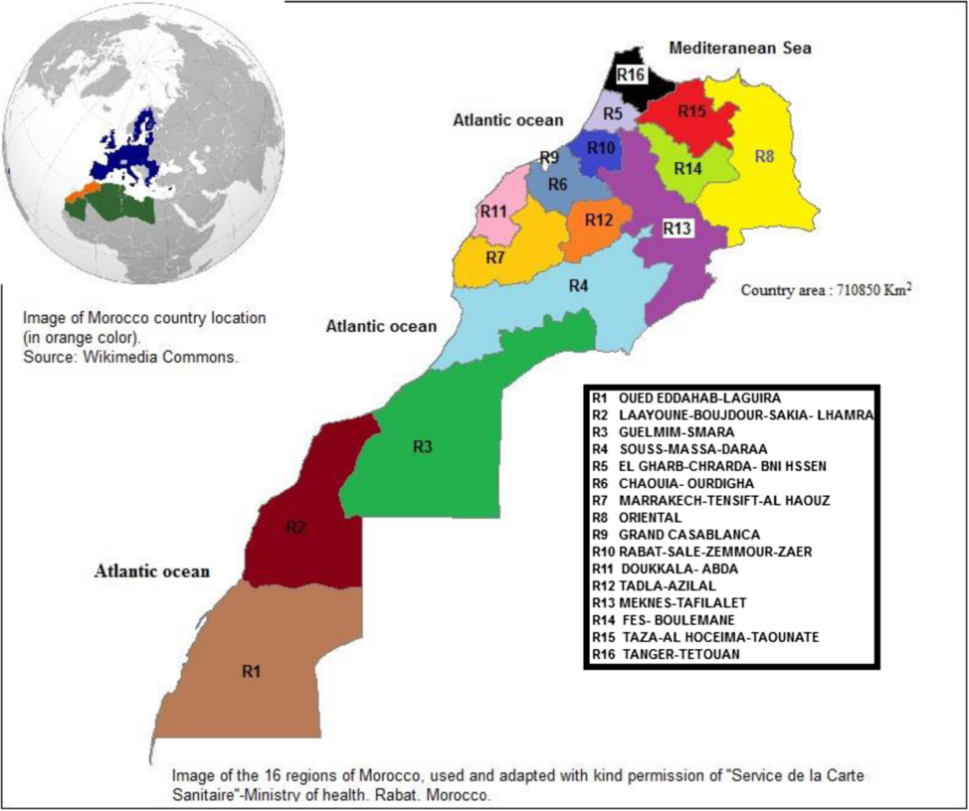
Map of Morocco, illustrating the 16 administrative regions. This figure illustrates the 16 administrative regions that make up Morocco

In this study, all of Morocco was considered (see Fig. 2). The country is located in North Africa and has an area of 710 850 km^2^ [9]. It is surrounded by the Mediterranean Sea in the north and the Atlantic Ocean in the west. The climate is Mediterranean with frequent precipitations in the northwestern part, and lower levels of precipitations in the east and south parts. In terms of topography, the Rif Mountains lie to the north, parallel to the Mediterranean Coast, while the Atlas Mountains (Middle Atlas, High Atlas, and Anti-Atlas ranges) stretch along from the Rif Mountains to the southwestern part of the country. Morocco has the highest mountains in North Africa: Jebel Toubkal in the High Atlas is 4 167 m high. It is believed that the Atlas Mountains are barriers to the transmission of human leishmaniasis from the east to the west. In the south, the desert of the Sahara prevails.

Morocco’s population was 30 079 000 in 2003, increasing to 32 951 000 in 2013 [4].

#### Regions and provinces/prefectures as units of analysis

Since the beginning of 2015, Morocco has been administratively subdivided into 12 regions. However, as the current study is interested only in the period of 2003–2013, the previous subdivisions [9], which comprised 16 regions (see Fig. 2), were considered. A region consists of provinces and prefectures, whereas a province or prefecture consists of communes. Secular time trends at the regional level covering the period of 2003–2013 were first examined. For spatial autocorrelation analysis, ‘province/prefecture’ rather than ‘commune’ was chosen as a unit of analysis. The reason behind this is that more data, in a range of covariates, are available at the province/prefecture level than at the commune level. Beside this, provinces are predominantly rural, whereas prefectures are predominantly urban, meaning that urbanization as a covariate could be studied. Furthermore, communes vary widely in terms of population size and may be characterized by a small unstable population; this could in turn affect the results of the spatial analysis.

### Source of data

#### Data related to leishmaniasis cases/incidence at the national level

Human leishmaniasis is an endemic disease that falls within the framework of a national Ministry of Health program related to vector-borne diseases [4]. The SSHHI-Ministry of Health provides both the total number of new detected leishmaniasis cases and the leishmaniasis incidence rate (combined CL and VL cases), by region, and by province/prefecture annually [4]. Published data were available only for the period of 2003 (edition 2004) to 2013 (edition 2014) [4]. Thus, the combination of CL and VL cases, forming the HLIR, is considered in the current study. No ethics approval was required for this study.

#### Data related to covariates

Covariates were chosen based on World Health Organization (WHO) guidelines [8] and data availability. Poverty rate and vulnerability rate as the two poverty indicators, the population density, and two degrees of urbanization as mentioned above (province versus prefecture) were chosen as the covariates.

Poverty rate data and vulnerability rate data, by province and prefecture, were freely accessible from “*Haut-Commissariat du Plan,”* which is the Moroccan Census Bureau, and available for the years 2004 and 2007 [10]. Because of the missing data for some provinces for the year 2004, only the year 2007 was considered for these covariates and the other study covariates. Population density was calculated based on population size data, which is freely available from the SSHHI-Ministry of Health, for the year 2007 [4].

### Statistical methods

**Fig. 3:**
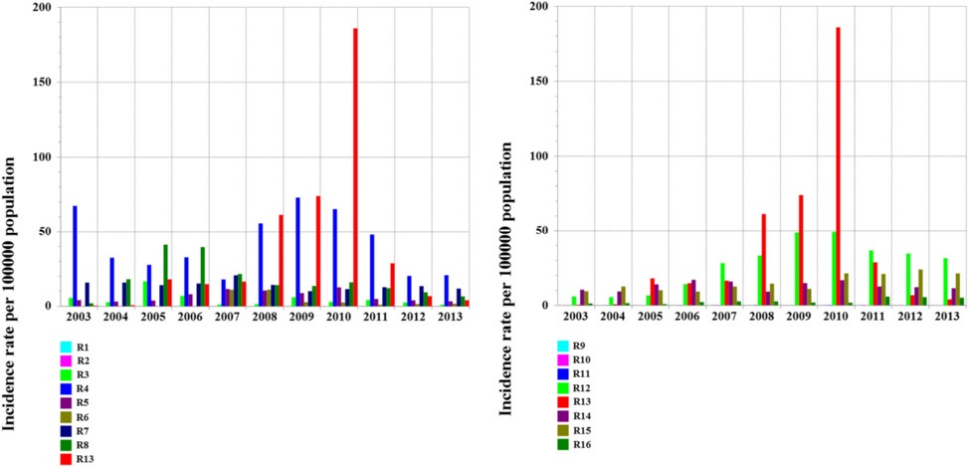
HLIR (CL and VL combined), by administrative region, Morocco, 2003–2013. This figure illustrates secular trends in human leishmaniasis in the 16 regions. The highest HLIR (186 per 100 000 population) was observed in R13 in 2010. A non-linear correlation (Gaussian curve) was observed in R12 (*P* = 0.01), a secular time increase was observed in R15 (*P* = 0.008) and R16 (*P* = 0.02), and a secular time decrease was observed in R7 (*P* = 0.04). No secular time variation was observed in the remaining administrative regions (*P* ≥ 0.05)

**Fig. 4:**
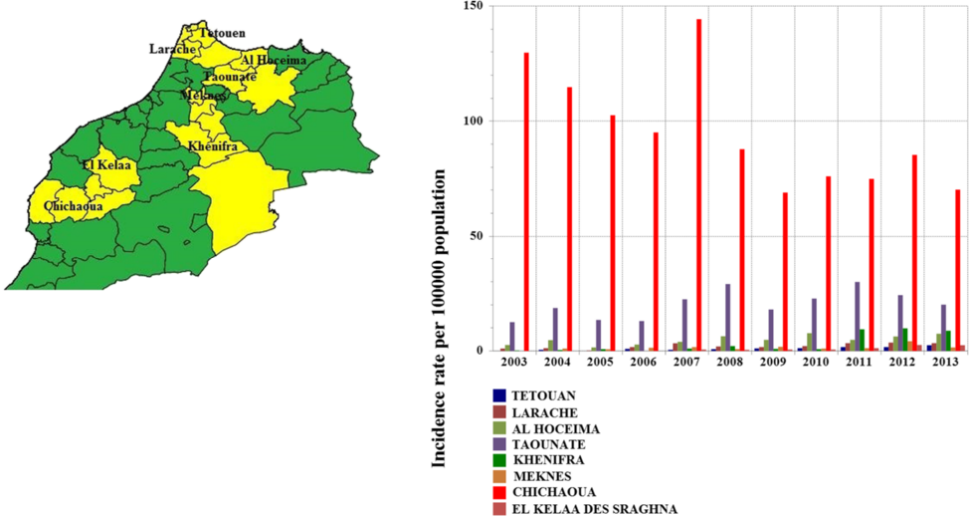
Location and secular trends in human leishmaniasis (CL and VL combined) incidence, in selected provinces in Morocco, 2003–2013. The increased incidence rate in R15 was evident in the Al Hoceima province (*P* = 0.008) and the Taounate province (*P* = 0.04). In R16, the rate increase was evident in the Larache province (*P* = 0.002) and the Tétouan province (*P* = 0.0003). In R13, it was evident in the Khenifra province (*P* = 0.008) and the Meknes province (*P* = 0.03). The decreased incidence rate in R7 was evident in the Chichaoua province (*P* = 0.006), even though an increase was observed in the El Kelaa province (*P* = 0.0007). No secular time variation was observed in the uncited provinces (*P* ≥ 0.05)

To provide the annual poisson incidence rate estimate and poisson rate confidence interval of CL and VL (see Fig. 1), the incidence rate was estimated as the number of events observed (cases of CL or VL) divided by the time at risk of event during the observation period (2003–2013). For CL, a total of 44 810 events were observed [4]; the time spent at risk of event was 3 445 370 10^8^. For VL, a total of 1 480 events were observed [4]; the time spent at risk of event was 3,445370 10^8^. Variations in human leishmaniasis incidence over time by region (see Fig. 3) and by province/prefecture were examined (see Fig. 4): an approximate two-sided Kendall’s rank correlation test was conducted, the *p*-values of which are provided. To evaluate variation in the incidence rate from 2010 to 2013 in the Errachidia province (see Discussion section), a Kendall’s rank test was calculated in exact form since sample size was too small. The HLIRs were 736.3, 151.4, and 12.9 per 100 000 population in 2010, 2011, 2012, respectively [4].

These statistical methods were conducted using the StatsDirect statistical software version 3.0.124 (StatsDirect Ltd., Cheshire, UK).

### Spatial autocorrelation analysis

#### Data preparation

A free-license shapefile map of Morocco, comprising only 47 provinces/prefectures, was downloaded [11] and modified with permission from the GADM databases of Global Administrative Areas [11]: the south part of Morocco was missing, thus a ruster image of the entire country, with 59 administrative subdivisions, was inputted and georeferenced. To add the missing provinces and prefectures that constitute the south part of Morocco, raster data was digitalized. This provided a shapefile map of Morocco with 59 provinces/prefectures. Attributes-related data, including population size for the year 2007, area size for each province and prefecture, and population density for the year 2007, were added/calculated. Urbanization was evaluated as a dummy variable.

According to the Moroccan Census Bureau, during the 11-study period (2003–2013), 15 of the 59 provinces/prefectures have been split each into two provinces, which led the SSHHI-Ministry of Health to provide HLIR for more than 59 provinces [4]. The incidence rate had then to be calculated for each of the 15 provinces/prefectures. These provinces/prefectures are Ouarzazate (split into Ouarzazate and Tinghir), Tiznit (split into Tiznit and Sidi Ifni), Kenitra (split into Kenitra and Sidi Slimane), Settat (split into Settat and Berrechid), El Kelaa (split into El Kelaa and Rhamna), Berkane (split into Berkane and Taourirt), Nador (split into Nador and Driouch), Casablanca (represents All Great Casablanca except for Mohammadia), El Jadida (split into El Jadida and Sidi Bennour), Safi (split into Safi and El Youssoufia), Beni Mellal (split into Beni Mellal and Fquih Ben Saleh), Khenifra (split into Khenifra and Midelt), Taza (split into Taza and Guercif), Tétouan (split into Tétouan and M’diq), and Sidi Kacem (split into Sidi Kacem and Ouazzane). The incidence rate in each of the 15 provinces/prefectures was calculated as the sum of cases of leishmaniasis in the two separate provinces/prefectures divided by the sum of population sizes in these two separate provinces/prefectures. All the required data on leishmaniasis cases and the popuation size were provided by SSHHI-Ministry of Health [4]. An incidence data file including the 59 provinces/prefectures and the study years was then created and joined with the already existing shapefile map of Morocco to produce an incidence shapefile map for the period of 2003 to 2013. Covariates data for the year 2007 were then joined with the last produced shapefile map. Spatial treatment was performed using the QGIS software version 2.0.1 ‘Dufour’ (Free Software Foundation, Inc., Boston, USA.

#### Spatial analysis

**Fig. 5:**
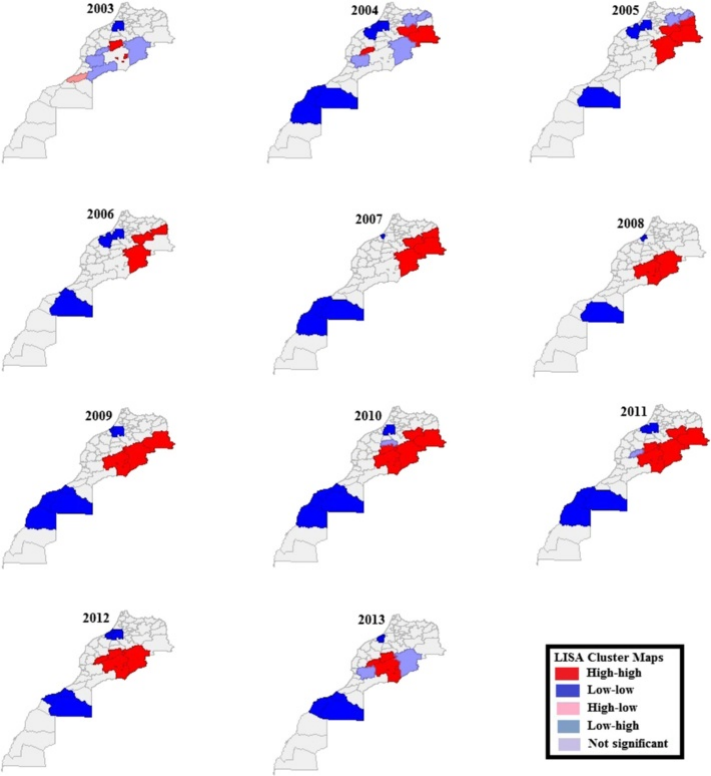
LISA cluster maps of human leishmaniasis incidence, Morocco, 2003–2013. The LISA indicated the presence or absence of significant spatial clusters or outliers for each province/prefecture. Significant clustering was seen from 2005 to 2013, during which the Errachidia province was considered to be a permanent ‘hot spot’ of human leishmaniasis (local Moran’s I highly significant *P* = 0.001). It seemed to be the main location from where leishmaniasis spread: from 2003 to 2007, high incidence clustering was observed in the north neighboring provinces (Figuig, Jrada, and Boulemane); in 2008, high incidence clustering disappeared from the north neighboring provinces and appeared in the south neighboring provinces (Ouarzazate and Zagora). From 2009, high incidence clusters again spread both north and south of the Errachidia province. Clusters disappeared in the north neighboring provinces in 2012 (showing a decrease in the Global Moran’s I statistic = 0.2993; *P* = 0.002), whereas they further spread, from 2010 to 2013, from the aforementioned south neighboring provinces all the way to southwestern neighboring provinces, such as Azilal and El Haouz. In 2013, a low incidence cluster surrounded by high incidence clusters was observed in the Errachidia province, explaining the further decrease in Global Moran’s I (Global Moran’s I statistic = 0.2491; *P* = 0.004). Clusters of low leishmaniasis incidence were observed in the southern part (Tan Tan, Tarfaya, Laayoun, Boujdour, and Essmara provinces), and in the northwestern part (Khemisset, Settat, Benslimane, Skhirat and Salé provinces, and Rabat prefecture) of the country

**Fig. 6:**
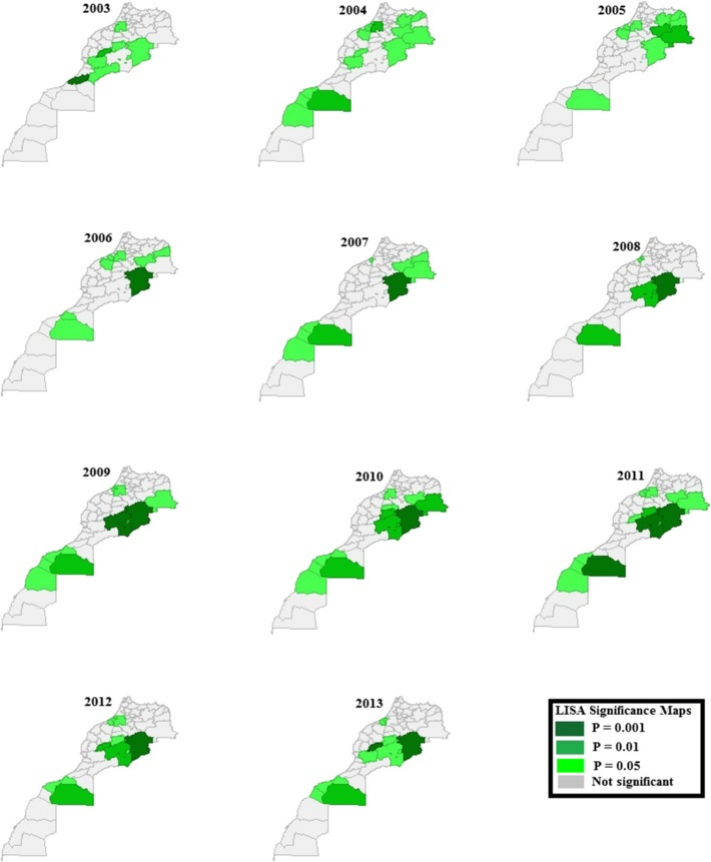
LISA significance maps of human leishmaniasis incidence, Morocco, 2003–2013

To examine spatial patterns and spatial diffusion, the exploratory spatial data analysis approach [12–17] was applied. GeoDa software version 1.6.7.9, March 2015, developed by Luc Anselin (ASU, GeoDa Center for Geospatial Analysis and Computation, Arizona, USA) was used for this purpose. First, a contiguity weight file at the province/prefecture level was created: queen contiguity, which defines spatial neighbors as those areas with shared borders and vertices, was chosen. Global Moran’s I statistics (see Table 2) were determined for each of the 11 study years (2003–2013). Local indicators of spatial association (LISA) showing the presence or absence of significant spatial clusters or outliers for each province/prefecture are shown by cluster maps (see Fig. 5). LISA significance maps are also provided (see Fig. 6).

**Table 2:** Global Moran’s *I* statistics : Human Leishmaniasis Incidence Rates (Empirical Pseudo-Significance Based on 999 Random Permutations)

Year	Global Morans’ *I* statistic	Pseudo-significance P (statistically significant if *P* < 0.05)
2003	- 0.0075	0.191
2004	0.0867	0.111
2005	0.2844	0.006
2006	0.2013	0.012
2007	0.2100	0.009
2008	0.3599	0.002
2009	0.3657	0.002
2010	0.4605	0.002
2011	0.5886	0.001
2012	0.2993	0.002
2013	0.2491	0.004

To test the covariates’ influence on leishmaniasis incidence, a spatial regression analysis (ordinary least squares) was carried out (see Table 3).

**Table 3:** Ordinary Least Squares (OLS) regression of human leishmaniasis incidence rates, provinces and prefectures, Morocco, 2007

Variables	Coefficient	t-Statistic	p-Value
Intercept	23.358	0.780	0.439
Province/prefecture	−7.998	−0.527	0.601
Vulnerability rate	−2.948	−1.938	0.058
Poverty rate	5.741	3.870	0.0003
Population density	2.646	0.064	0.949
	Adjusted R-squared: 0.2255	F-statistic: 5.221	p-Value (F-statistic): 0.001

*N* = 59 units of analysis

## Results

### Secular time trends in leishmaniasis incidence in the 16 Moroccan regions

The highest HLIR (186 per 100 000 population) was observed in region 13 (R13) in 2010 (see Fig. 3). A non-linear correlation (Gaussian curve) was observed in R12 (*P* = 0.01), a secular time increase was observed in R15 (*P* = 0.008) and R16 (*P* = 0.02), and a secular time decrease was observed in R7 (*P* = 0.04). No secular time variation was observed in the remaining administrative regions (*P* ≥ 0.05) (see Fig. 3).

The increased incidence rate in R15 was evident in the Al Hoceima province (*P* = 0.008) and the Taounate province (*P* = 0.04). In R16, the rate increase was evident in the Larache province (*P* = 0.002) and in the Tétouan province (*P* = 0.0003). In R13, it was evident in the Khenifra province (*P* = 0.008) and the Meknes province (*P* = 0.03). The rate decrease in R7 was evident in the Chichaoua province (*P* = 0.006), even though an increase was observed in the El Kelaa province (*P* = 0.0007). No secular time variation was observed in the uncited provinces (*P* ≥ 0.05). The yellow color in Fig. 4 shows provinces where the increased or decreased rate was evident.

### Spatial autocorrelation analysis and spatial diffusion

A total of 59 provinces and prefectures were considered for the spatial analysis. Moran’s I statistics over this period were examined. Dispersed high and low values were shown in 2003 (Global Moran’s I statistic = -0.0075), whereas random clustering was seen in 2004 (Global Moran’s I statistic = 0.0867; *P* = 0.111). Significant clustering was seen from 2005 to 2013 (see Table 2), during which period the Errachidia province was a permanent ‘hot spot’ of human leishmaniasis (local Moran’s I highly significant *P* = 0.001). It seemed to be the main location from where leishmaniasis spread: from 2003 to 2007, high incidence clustering was observed in the north neighboring provinces of the Errachidia province, including in Figuig, Jrada, and Boulemane; in 2008, high incidence clustering disappeared in the north neighboring provinces and appeared in south neighboring provinces, such as Ouarzazate and Zagora (see Figs. 5 and 6). From 2009, high incidence clusters again spread both north and south of the Errachidia province (see Figs. 5 and 6), disappearing in the north neighboring provinces in 2012 (showing a decrease in Global Moran’s I statistic = 0.2993; *P* = 0.002), but further spreading, from 2010 to 2013, in the aforementioned south neighboring provinces all the way to southwestern neighboring provinces, such as Azilal and El Haouz (see Figs. 5 and 6). In 2013, a low incidence cluster surrounded by high incidence clusters was observed in the Errachidia province, explaining the decrease in Global Moran’s I (Global Moran’s I statistic = 0.2491; *P* = 0.004).

Clusters of low leishmaniasis incidence were seen in the southern part of Morocco, including the Tan Tan, Tarfaya, Laayoun, Boujdour, and Essmara provinces, where incidence rates was as low as zero; and in the northwestern part of the country, including the Khemisset, Settat, Benslimane, Skhirat and Salé provinces, and the Rabat prefecture, where incidence rates were less than 0.6 per 100,000 population (see Figs. 5 and 6).

### Spatial regression

The spatial regression analysis (see Table 3) showed that only poverty had an effect on leishmaniasis incidence (*P* = 0.0003). To further examine the effect of this covariate, a LISA cluster map of poverty rates (see Fig. 8; Image 1), a LISA significance map of poverty rates (see Fig. 8; Image 2), a histogram of poverty rates (see Fig. 8, Images 3 and 4) for the year 2007, a bivariate cluster map (see Fig. 8; Image 5), illustrating the relationship between leishmaniasis incidence and poverty, and a corresponding bivariate significance map (see Fig. 8; Image 6) for the same year, were all drawn up. A box plot showing leishmaniasis incidence rate data for the year 2007 (see Fig. 8; Image 7) was also provided.

The northeastern part of the country showed high incidence rate clusters associated with high poverty rate clusters (see Fig. 8; Images 5 and 6). Both the yellow grids in the histogram (see Fig. 8; Image 3) and the four yellow points in the box plot (see Fig. 8; Image 7) correspond to significant clusters of high leishmaniasis incidence, as shown in Fig. 5, i.e. in the Errachidia, Figuig, Boulemane, and Jrada provinces. Likewise, the yellow grids in the histogram (see Fig. 8; Image 4) correspond to significant clusters of low leishmaniasis incidence, as shown in Fig. 5, i.e. in the Tan Tan, Tarfaya, Laayoun, and Essmara provinces.

## Discussion

In this study, spatial patterns and secular trends in human leishmaniasis incidence in Morocco between 2003 and 2013 were examined, based on the only available published national data, from the open access files of the Ministry of Health. No similar study, identifying spatial autocorrelation and trends in human leishmaniasis, was previously conducted in Morocco, which would have allowed the findings of this research to be compared with previous findings. According to Fig. 1, the data on human leishmaniasis presented in the current study shows that CL incidence rates clearly dominate: there is a perfect overlap between the HLIR (CL and VL combined) and CL incidence rates (see Fig. 1). Cases and incidence of VL remain low and negligible compared to CL cases and incidence.

It is interesting to note that provinces where increased or decreased rate was evident (see Fig. 4), none of these provinces showed clustering of leishmaniasis incidence (see Figs. 5 and 6). Anthroponotic CL cases of the *L. tropica* strain have been reported in these provinces [6], however, further studies and new scientific approaches including risk assessment and GIS tools are required to identify risk factors related to leishmaniasis secular time trends, particularly in terms of secular increases.

Vector species and reservoirs identified as being responsible for human leishmaniasis in Morocco are shown in Table 1. A recent entomological study [18] carried out in five sparce areas in Morocco, different in terms of altitude and bioclimate, showed that *Phlebotomus sergenti*, *Ph. perniciosus*, *Ph. longicuspis*, *Ph. papatasi*, and *Syrnola minuta* were all present in the five areas, but species predominance varied. No area in the south of Morocco was chosen in this just described study [18]. Entomological studies have previously been carried out in Moroccan provinces/prefectures, including Sefrou [19], Marrakech [20], Taza [21], and Moulay Yacoub [22], in all of which neither a cluster presence nor a trend variation in leishmaniasis incidence was observed during the period in question in the current study. No similar study has been undertaken in Beni Mellal or Taroudant, where clustering was observed in this study in 2010 and 2013, respectively (see Fig. 5, light blue color). This highlights the importance of conducting spatial analysis as to determine where conducting studies and implementing vector control measures should be a priority. In spite of this, findings of the current study when comparing them to studies undertaken in provinces suspected to have leishmaniasis incidence may be informative. In Chichaoua, the highest HLIRs (CL and VL combined) were observed (see Fig. 4), and a high seroprevalence of canine leishmaniasis as well as low human VL incidence rates were reported [23]. Similar fluctuations and same activity period of *Phlebotomus perniciosus* and *Phlebotomus longicuspis* were observed in Chichaoua as in the Sefrou province [24]. However, no cluster or trend was observed in Sefrou, whereas a secular decrease in incidence rates were observed in Chichaoua (see Fig. 4). High seroprevalences of canine leishmaniasis and low human VL incidence rates were also reported in Marrakech, El Haouz, and Azilal [23], however, in the current study, in none of these provinces was a trend variation observed, and significant clustering was only observed from 2010 to 2013 in Azilal and El Haouz, as a result of a potential diffusion of zoonotic CL from the northeastern provinces (see Fig. 5).

Global Moran’s I statistics were examined in an attempt to understand leishmaniasis spatial diffusion in the country in the 11-year study period. There was evidence of the presence of significant spatial clusters from 2005 to 2013 (see Table 2). Spatial clusters of high leishmaniasis incidence were observed in the northeastern parts of the country, whereas low incidence rates were observed in some northwestern and southern parts of the country. No evidence of spatial clustering was observed in the remaining parts of the country (see Figs. 5 and 6). During the period during which significant spatial clusters (2005–2013) were observed, the Errachidia province was continuously considered a ‘hot spot’ of leishmaniasis, whereas the neighboring provinces of Jrada, Figuig, Boulemane, Errachidia, Ouarzazate, Zagora, Azilal, and El Haouz had intermittent significant spatial clusters over time. It appears that leishmaniasis spread from the Errachidia province to the north, south, and west neighboring provinces. The distribution of different forms of leishmaniasis and species of leishmania throughout the country is documented elsewhere [6]. According to previous studies [6, 25, 26], the northeastern part of Morocco, comprising the provinces of Jrada, Figuig, Boulemane, Errachidia, Ouarzazate, and Zagora, in which a highly significant clustering was observed in the current study (see Figs. 5 and 6), are foci of zoonotic CL caused by *L. major* that is transmitted by *P. papatasi*, with *Meriones shawi grandis* as the main reservoir host (see Table 1). Since these provinces neighbor Errachidia and are similar in terms of reservoir host types, vector types, leishmania species, seasonal weather variations, temperatures, humidity, warming, drought, and nationally adopted leishmaniasis-related control measures, one may wonder why highly significant clustering of high leishmaniasis incidence was continuously observed in the Errachidia province during the study period, whereas clustering appeared and disappeared over time in the neighboring provinces.

In a previous study [27], *Meriones shawi* was targeted in the Errachidia province, using strychnine-poisoned wheat baits, from 2010 to 2012 [27]. The previous question is raised again: we know that despite rodent control measures in the Errachidia province, highly significant clustering persisted in the province (see Figs. 5 and 6), yet significant decreases in the incidence rate (736.3, 151.4, and 12.9 per 100,000 population in 2010, 2011, 2012, respectively [4]) were observed (Kendall’s test in exact form, performed in the current study: *P* < 0.0001). In 2013, the incidence rate was 3.2 per 100,000 population [4] in the Errachidia province, which may suggest that the rodent control measures were successful, and may explain the presence of significant clusters of low leishmaniasis incidence, i.e. in the Errachidia province, as compared to those of high leishmaniasis incidence, i.e. in the Ouarzazate, Zagora, Azilal, and El Haouz provinces (see Fig. 5).

**Fig. 7:**
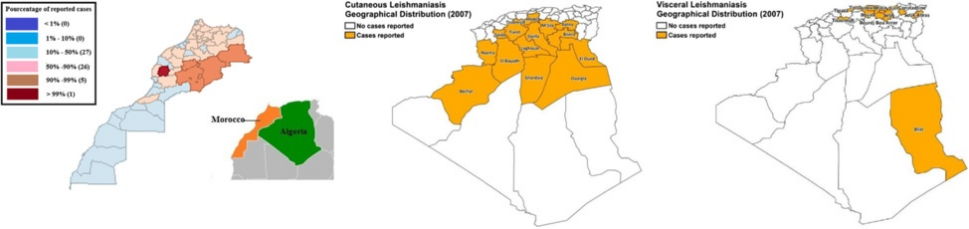
**a** Geographical distribution of human leishmaniasis cases, Algeria, 2007. **b** Geographical distribution of human leishmaniasis cases, Morocco, 2007. These figures show the geographical distribution of human leishmaniasis cases in Algeria and Morocco, respectively. A potential spread of CL from the Béchar province in Algeria to the Errachidia province in Morocco is suspected

Algeria is a neighboring country of Morocco and the second largest focus of CL in the world after Afghanistan [28]. Looking at leishmaniasis incidence in Algeria during the study period (2003–2013) showed that CL cases in this country highly outnumbered those in Morocco [7, 28, 29]: CL was endemic in 40 of the 48 provinces [7], there is a lack of legislation regarding vector control, and the spraying of poisons near houses to kill *Meriones shawi* is not allowed [7] in Algeria. Data on leishmaniasis incidence distribution in Algeria between 2003 and 2013 are scarce, however, figures depicting the geographical distribution of CL and VL cases in Algeria in 2007 were available [28]. They were compared with a percentile map of leishmaniasis cases in Morocco, using the data of the same year (2007). A look at the two maps (see Fig. 7a and b) suggests a potential spread of CL, particularly from the Béchar province in Algeria, where there is a high risk of leishmaniasis [30], to the Errachidia province.

Clusters of low leishmaniasis incidence were seen in the southern part of Morocco, including in the Tan Tan, Tarfaya, Laayoun, Boujdour, and Essmara provinces, where incidence rates were as low as zero, which is consistent with findings of a study carried out in the 1990s that reported a scarcity of vector species and an absence of leishmaniasis cases [31]. Anthroponotic CL cases and zoonotic CL cases were reported in the Guelmim and Taza provinces, respectively [4, 6], however, no significant clustering was identified in these provinces in the current study.

In Morocco, CL, due to *L. tropica* reported to be transmitted by the sandfly *P. sergenti* (see Table 1), appears to be anthroponotic and occurs between Tadla and Agadir provinces, and in the ‘subhumid’ climate zone north and west of the High Atlas [1, 6]. However, the WHO-EMRO reported that, in Morocco, studies have shown a discrepancy between parasites in humans and vectors, and that this strongly indicates the possibility of an unknown reservoir host [1]. It has been reported that *L. tropica* [32, 33] has been isolated from both the domestic dog and the black rat in other countries; in some rural areas of Morocco, domestic dogs were also found to be infected with *L. tropic*a. However, if an unknown reservoir host was predominatly present in Moroccan provinces where CL due to *L. tropica* were reported [6], an evidence of clustering in these provinces would have been observed in the current study, which was not the case (see Fig. 5). In the northwestern part of the country, including the Khemisset, Settat, Benslimane, Skhirat and Salé provinces, and the Rabat prefecture, significant clusters were observed when leishmaniasis incidence rates were less than 0.6 per 100 000 population. Higher incidence rates (19 per 100 000 population in 2007 and 2008) were reported in the Settat province [4], however, the LISA was not significant. Further studies/investigation would yield more information.

**Fig. 8:**
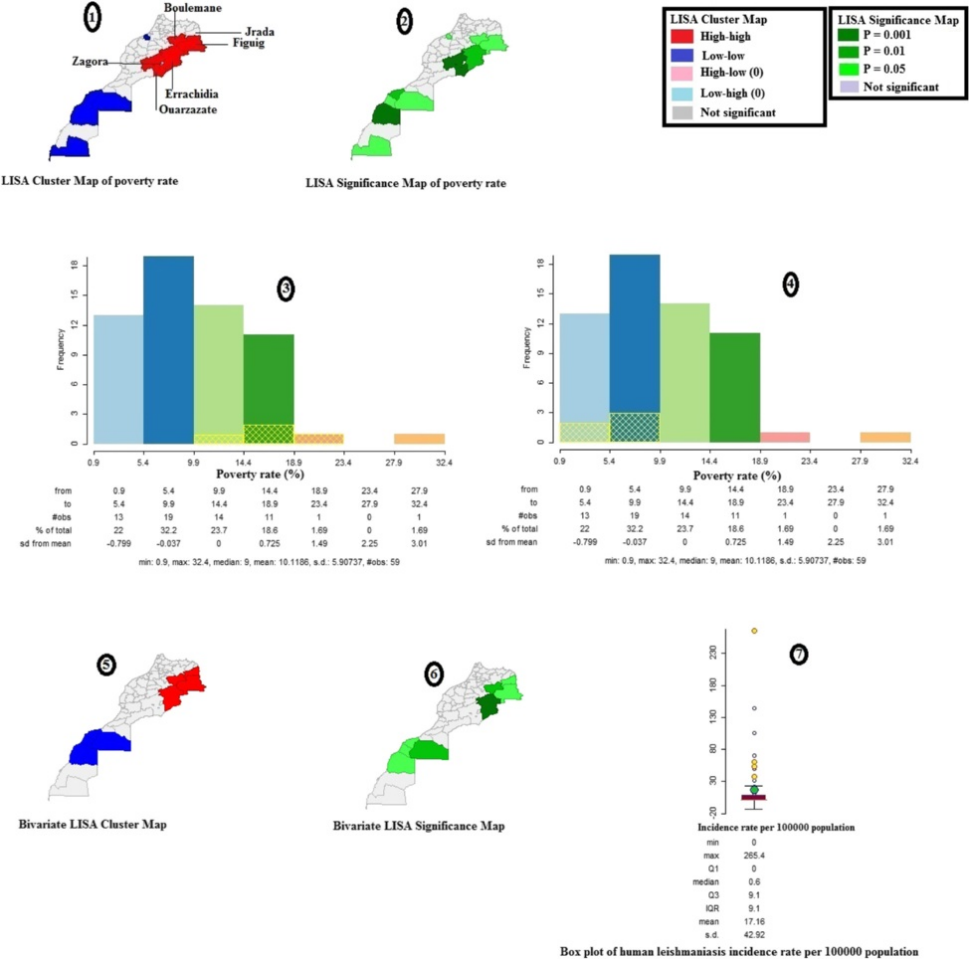
Association between human leishmaniasis incidence rate and poverty rate, Morocco, 2007. High incidence clusters in the northeastern part of the country were associated with high poverty clusters (Images 5 and 6). Both the yellow grids in the histogram of poverty (Image 3) and the four yellow points in the box plot (Image 7) correspond to significant clusters of high leishmaniasis incidence (Errachidia, Figuig, Boulemane, and Jrada provinces) shown in Fig. 5. Likewise, the yellow grids in the histogram of poverty (Image 4) correspond to significant clusters of low leishmaniasis incidence shown in Fig. 5 (Tan Tan, Tarfaya, Laayoun, and Essmara provinces)

To determine other potential factors that may affect leishmaniasis incidence in Morocco, the poverty rate, the vulnerability rate, population density, and urbanization at the province/prefecture level were examined. Only poverty was found to have a significant effect on leishmaniasis incidence, contributing only 23 % to this (see Table 3: Adjusted R-squared = 0.226). High-high leishmaniasis incidence rate clusters corresponding to Errachidia, Figuig, Jrada, and Boulemane provinces were associated with high-high poverty rate clusters and vice-versa (see Fig. 8).

This study had limitations. Data related to human CL and VL cases, separately published by province/prefecture or by region, are not available. However, the distribution of different forms and species of leishmaniasis throughout the country is documented elsewhere [6], which helped in the interpretation of the findings of this study. Also, data on covariates and leishmaniasis were not known at the level of the individual : in this study, the unit of analysis was the group.

The strength of this study is the description of the spatial patterns of human leishmaniasis incidence over an 11-year period in the whole of Morocco, including in the south of it where related information was lacking, the identification of provinces/prefectures showing either secular time increase or significant clustering, and the examination of some covariates that may influence human leishmaniasis incidence. All this was done by means of not-well-known scientific approaches in Morocco, such as exploratory spatial data analysis. Findings indicate the need for different stakeholders to address leishmaniasis in Morocco.

## Conclusion

New information on leishmaniasis incidence in Morocco was provided. This study highlights the importance of conducting spatial analysis as to determine where conducting studies on leishmaniasis and implementing vector and rodent control measures should be a priority. Further studies and new scientific approaches including risk assessment and GIS tools would identify underlying risk factors related to leishmaniasis; studying small areas and relying on data at the level of the individual, provided necessary data are available, would yield more information on risk factors that may affect leishmaniasis incidence in Morocco. This may be considered in future studies related to leishmaniasis. Poverty contributes to leishmaniasis in Morocco, which requires joint efforts of many stakeholders.

## Ethics and consent

Ethical approval for this study is not applicable as the data are publicly available.

## Additional file

Additional file 1: Multilingual abstracts in the six official working languages of the United Nations. (PDF 502 kb)

## Abbreviations

CL: Cutaneous leishmaniasis
HLIR: Human leishmaniasis incidence rate
LISA: Local indicator of spatial association
R: Region
SSHHI: Service of Studies in Health and Health Information
VL: Visceral leishmaniasis
WHO: World Health Organization
