# Supplementary material for: Spatial patterns and secular trends in human leishmaniasis incidence in Morocco between 2003 and 2013
Source: Infect Dis Poverty. 2016 May 11;5:48. doi: 10.1186/s40249-016-0135-8 (PMC4863334; doi:10.1186/s40249-016-0135-8)
Supplement: Additional file 1: — Multilingual abstracts in the six official working languages of the United Nations. (PDF 502 kb) [file 40249_2016_135_MOESM1_ESM.pdf]

Translation of the abstract into the six official working languages of the United Nations

أنماط مكانية وتوجهات مدنية في حدوث داء الليشمانيات في المغرب ما بين عامي 2003 و2013

مينا صادق

ملخص

خلفية: تم إجراء عدد قليل من الدراسات عن الأنماط المكانية والتوجهات المدنية في حدوث داء الليشمانيات البشرية في المغرب. وتهدف هذه الدراسة إلى فحص الأنماط المكانية والتوجهات المدنية المرتبطة بمعدل حدوث داء الليشمانيات البشرية (HLIR) على مستوى المقاطعة/ الولاية فيما بين عامي 2003 و2013 في المغرب.

الطرق: تم فقط استخدام بيانات الدولة المنشورة والمتوافرة بشأن معدل حدوث داء الليشمانيات البشرية (HLIR) فينا بين عامي 2003 و2013، من ملفات وزارة الصحة المسموح الوصول إليها. تم فحص التوجهات المدنية باستخدام معامل ارتباط تصنيف كندل. كما تم إجراء تحليل بيانات مكاني استكشافي لفحص الترابط التلقائي (قياس Moran's I الشامل ومؤشر محلي للارتباط المكاني [LISA])، والانتشار المكاني على مستوى المقاطعة/ الولاية. كما تم اختبار تأثير العديد من العوامل (معدل الفقر، معدل القابلية للإصابة، الكثافة السكانية والتمدن) على معدل حدوث داء الليشمانيات البشرية (HLIR) باستخدام الانحدار المكاني(مربعات صغرى عادية).

النتائج: على مستوى الدولة، لم يتم ملاحظة تغير مدني. يقدر توزيع بواسون السنوي للوقائع بأنها كانت 13 لكل 100.000 نسمة (-95% CI- 12.9 13.1) بالنسبة لداء ليشمانيات كتانيوس (CL) و0.4 لكل 100.000 نسمة (95% CL-0.4-0.5) لداء الليشمانيات الأمعائي (VL). وتقوم البيانات المتوافرة عن معدل حدوث داء الليشمانيات البشرية (HLIR) على مزج حالات داء ليشمانيات كتانيوس (CL) وداء الليشمانيات الأمعائي (VL)، ولكن، حيث أن حالات ليشمانيات كتانيوس (CL) تفوق في العدد حالات داء الليشمانيات الأمعائي (VL)، فإنه يمكن اعتبار أن معدل حدوث داء الليشمانيات البشرية (HLIR) هو معدل حدوث حالات ليشمانيات كتانيوس (CL). على مستوى المقاطعة، تم ملاحظة تزايد المعدل المدني في الحالات في الحُسيمة (p=0.03) والكيلا (p=0.0007)، بينما تم ملاحظة الانخفاض المدني فقط في مقاطعة شيشاوا (p-=0.006).

وبرغم أن معدل الازدياد أو الانخفاض كان واضحاً في تلك المقاطعات، فإن أيا منها لم يُظهر وجود تجمع عنقودي لحدوث داء الليشمانيات. تم تحديد تجمعات عنقودية مكانية ملحوظة لوقوع عدد كبير من حالات داء الليشمانيات في الجزء الشمالي الشرقي من المغرب، بينما كانت التجمعات العنقودية المكانية الأقل وقوعاً من حالات داء الليشمانيات كانت في باقي أجزاء البلاد. تم مشاهدة حدوث تجمعات عنقودية ملحوظة فيما بين عامي2005 إلى 2013، حيث كانت مقاطعة الرشيدية خلال تلك الفترة "نقطة مشتعلة" دائمة، وقد زاد مؤشر Moran'I الشامل من 0.2844 (p=0.006) في عام 2005 إلى 0.5886 (p=0.001) في عام 2011، وانخفض إلى 0.2491 (p=0.004) في عام 2013. وقد وُجِد أن الفقر وحده هو ما يؤثر على معدل حدوث داء الليشمانيات البشرية (HLIR) (p=0.0003)، مساهماً بنسبة 23% في ذلك (R – التربيعية المعدلة = 0.226).

الاستنتاج: تم تحديد المحليات تُزهر إما زيادة مدنية في داء الليشمانيات البشرية أو تجمع عنقودي ملحوظ، مما قد يرشد صُناع القرار بشأن أين ما يجب تخصيص المعونات وتنفيذ إجراءات السيطرة بصورة ملائمة. كما يجب الحث على إجراء الأبحاث من أجل تنفيذ المزيد من الدراسات التي تقوم بالتركيز على تلك المحليات.

Translated from English version into Arabic by REHAM HUSSIEN, through

TRANSLATORS WITHOUT BORDERS

2003 年至 2013 年摩洛哥人利什曼病发病率的空间模式和长期趋势

Mina Sadeq

摘要

引言：很少研究关注摩洛哥人利什曼病的空间模式和长期趋势。本研究分析 2003 年至 2013 年摩洛哥省/市级水平人利什曼病发病率的空间模式和长期趋势。

方法：仅利用卫生部公开文件中业已发表的 2003 年至 2013 年的人利什曼病发病率数据。采用 Kendall 秩相关分析长期趋势。采用探索性空间数据分析法来了解省/市级水平的空间自相关（全局莫兰指数和空间关联局域指标）和空间扩散。采用空间回归（普通最小二乘回归）分析多种协变量（贫困率、脆弱指数、人口密度和城镇化）对人利什曼病发病率的影响。

结果：未在国家水平发现长期趋势。皮肤利什曼病和内脏利什曼病年发病率 Poisson 估计分别是 13/100 000（95%置信区间：12.9–13.1）和 0.4/100 000（95%置信区间：0.4–0.5）。可获得的人利什曼病发病率数据基于皮肤利什曼病和内脏利什曼病病例的合计。因为皮肤利什曼病病例远远高于内脏利什曼病病例，所以人利什曼病发病率数据可以近似为皮肤利什曼病发病率数据。在 Al Hoceima (*P*=0.008)、Taounate (*P*=0.04)、Larache (*P*=0.002)、Tétouan (*P*=0.0003)、Khenifra (*P*=0.008)、Meknes (*P*=0.03)和 El Kelaa (*P*=0.0007) 发现省级水平的长期增加趋势，而仅在 Chichaoua 省 (*P*=0.006)发现长期降低趋势。尽管以上省感染率出现明显的上升或下降，但是均未表现利什曼病发病率的聚集性。显著的利什曼病高发病率空间聚集性发生在摩洛哥东北部，而利什曼病低发病率空间聚集性发生在摩洛哥西北部和南部地区，其他地区则表现为空间自由性。2005 年到 2013 年表现为明显的聚集性，该段时间 Errachidia 省一直是“热点”地区。全局莫兰指数从 2005 年的 0.2844 (*P*=0.006)增加到 2011 年的 0.5886 (*P*=0.001) ，后又下降到 2013 年的 0.2491 (*P*=0.004)。仅发现贫困率(*P*= 0.0003)对人利什曼病发病率有影响，大约占 23%（调整 R^2 为 0.226）。

结论：局部发现人利什曼病的长期趋势或显著的聚集性，可指导决策以合理分配资金和采取控制措施。同样亟待加强研究对上述局部地区作进一步分析。

Translated from English version into Chinese by Qian Menbao, through

CHINESE CENTER FOR DISEASE CONTROL AND PREVENTION
NATIONAL INSTITUTE OF PARASITIC DISEASES

Répartitions spatiales et tendances à long terme dans l'incidence de la leishmaniose humaine au Maroc entre 2003 et 2013

Mina Sadeq

Résumé

Contexte : peu d'études ont été réalisées au Maroc sur les répartitions spatiales ou les tendances à long terme de la leishmaniose humaine. La présente étude avait pour objectif d'examiner les répartitions spatiales et les tendances associées au taux d'incidence de la leishmaniose humaine (HLIR) au niveau provincial/préfectural entre 2003 et 2013 au Maroc.

Méthodes : seules les données nationales relatives au HLIR entre 2003 et 2013 publiées par le Ministère de la Santé et mises à disposition sous la forme de fichiers en accès libre ont été utilisées. Des tendances à long terme ont été examinées à l'aide de la corrélation des classements de Kendall. Une analyse spatiale des données à des fins exploratoires a aussi été réalisée afin d'examiner l'autocorrélation spatiale (indice de Moran global et indicateur local d'association spatial [LISA]) ainsi que la diffusion spatiale au niveau provincial/préfectural. L'influence de divers covariables (taux de pauvreté, taux de vulnérabilité, densité de la population et d'urbanisation) sur le HLIR a été mesurée par l'intermédiaire d'une méthode de régression spatiale (méthode classique des moindres carrés).

Résultats : au niveau national, aucune variation à long terme n'a été observée. Les estimations du taux d'incidence annuel de Poisson s'élevaient à 13 pour 100 000 personnes (IC à 95 % = 12,9–13,1) pour la leishmaniose cutanée (CL) et à 0,4 pour 100 000 personnes (IC à 95 % = 0,4–0,5) pour la leishmaniose viscérale (VL). Les données

disponibles relatives au HLIR reposaient sur la combinaison des cas de CL et de VL, mais les cas de CL ayant totalement dépassé les cas de VL, il est possible de considérer le HLIR comme correspondant au taux d'incidence du CL. Au niveau provincial, une hausse à long terme du taux d'incidence a été observée à Al Hoceima (*p*=0,008), Taounate (*p*=0,04), Larache (*p*=0,002), Tétouan (*p*=0,0003), Khenifra (*p*=0,008), Meknès (*p*=0,03) et El Kelaa (*p*=0,0007), tandis qu'une diminution à long terme a été observée uniquement dans la province de Chichaoua (*p*=0,006). Même si une hausse ou une baisse du taux était évident dans ces provinces, aucune d'entre elles ne présentait un regroupement d'incidence de la leishmaniose. Des groupes spatiaux significatifs de forte incidence de la leishmaniose se trouvaient dans le nord-est du Maroc, tandis que des groupes spatiaux de faible incidence de la leishmaniose ont été observés dans certaines régions du nord-ouest et du sud du Maroc. Une répartition aléatoire a été constatée dans les autres régions du pays. Un regroupement significatif a été observé de 2005 à 2013 et la province d'Errachidia constituait un « point chaud » permanent au cours de cette période. L'indice de Moran global a augmenté de 0,2844 (*p*=0,006) en 2005 à 0,5886 (*p*=0,001) en 2011 et a diminué à 0,2491 (*p*=0,004) en 2013. Nous avons déterminé que seule la pauvreté avait un effet sur le HLIR (*p*= 0,0003), contribuant à ce dernier à hauteur de seulement 23 % (R-carré ajusté = 0,226).

Conclusion : des localités affichant une hausse à long terme de l'incidence de la leishmaniose humaine ou un regroupement significatif ont été identifiées, ce qui peut guider la prise de décisions quant à l'allocation appropriée de fonds et à la mise en œuvre de mesures de lutte. Les chercheurs ont aussi encouragé la réalisation d'autres études se concentrant sur ces localités.

Translated from English version into French by eric ragu, through

TRANSLATORS WITHOUT BORDERS

Схемы распространения и долговременные тенденции заболеваемости лейшманиозом человека в Марокко в период с 2003 по 2013 гг.

Мина Садек

Краткое изложение

История вопроса: В Марокко было проведено очень незначительное количество исследований схем распространения и долговременных тенденций заболеваемости лейшманиозом человека. Данное исследование направлено на рассмотрение схем распространения и тенденций, связанных с коэффициентом заболеваемости лейшманиозом человека (КЗЛЧ) на уровне провинций/префектур в Марокко в период с 2003 по 2013 гг.

Методы: Были использованы только доступные опубликованные данные по стране по КЗЛЧ с 2003 по 2013 гг. из находящихся в открытом доступе файлов Министерства здравоохранения. Долговременные тенденции рассматривались с помощью коэффициента ранговой корреляции Кендалла. Также был проведен анализ данных схем распространения для рассмотрения пространственной автокорреляции (Global Moran's I и локальный индикатор пространственной связи (ЛИПС)) и пространственного проникновения на уровне провинций/префектур. Воздействие различных ковариантов (уровень бедности, коэффициент восприимчивости, плотность населения и урбанизация) на КЗЛЧ было протестировано путем

пространственной регрессии (обычного метода наименьших квадратов).

Результаты: На уровне страны не наблюдалось долговременных вариаций. Предварительная оценка годового коэффициента заболеваемости по Пуассону была 13 на 100 000 человек (95% CI = 12,9–13,1) для кожного лейшманиоза (КЛ) и 0,4 на 100 000 человек (95% CI = 0,4–0,5) для висцерального лейшманиоза (ВЛ). Доступные данные для коэффициента заболеваемости лейшманиозом человека основывались на общих данных для кожного и висцерального типа болезни, но поскольку количество заболеваний КЛ значительно превышает ВЛ, этот коэффициент может считаться коэффициентом заболеваемости кожным лейшманиозом. На уровне провинций долговременное повышение коэффициента заболеваемости наблюдалось в Эль-Хосейме (p=0,008), Таоунате (p=0,04), Эль-Араише (p=0,002), Тетуане (p=0,0003), Хенифре (p=0,008), Мекнесе (p=0,03) и Эль-Келаа (p=0.0007), а долговременное снижение – только в провинции Шишауа (p=0,006). Хотя повышение или снижение коэффициента в этих провинциях было очевидным, ни в одной из них не наблюдалось территориального сосредоточения заболеваемости. Значительное территориальное сосредоточение высокой заболеваемости наблюдалось в северо-восточной части Марокко, а низкой заболеваемости – на северо-западе и на юге станы. В остальных частях страны территориальное сосредоточение было нестабильным. Значительное сосредоточение наблюдалось с 2005 по 2013 гг., и в этот период постоянной «горячей точкой» была провинция Эррашидия. Global Moran’s I повысился с 0,2844 (p=0,006) в 2005 г. до 0,5886 (p=0,001) в 2011 г. и понизился до 0,2491 (p=0,004) в 2013 г.. Было установлено, что только уровень бедности влиял на КЗЛЧ (p= 0,0003), составляя всего 23% (скорректированный R-квадрат = 0,226).

Заключение: В ходе исследования были выявлены территории, на которых наблюдалось долговременное повышение заболеваемости лейшманиозом человека или его значительное сосредоточение, что может помочь в принятии решений о том, где именно необходимо принимать контрольные меры и куда следует направлять средства. Рекомендуется также проводить дальнейшие исследования для этих территорий.

Translated from English version into Russian by Elena McDonnell, through

TRANSLATORS
WITHOUT BORDERS

Patrones espaciales y tendencias de largo plazo de la incidencia de la leishmaniasis humana en Marruecos entre 2003 y 2013

Mina Sadeq

Resumen

Antecedentes: En Marruecos se han realizado pocos estudios sobre patrones espaciales o tendencias en el largo plazo de la leishmaniasis humana. El presente estudio se realizó con la finalidad de examinar los patrones espaciales y las tendencias de largo plazo asociados a la tasa de incidencia de la leishmaniasis humana (HLIR por sus iniciales en inglés) a nivel de las provincias/prefecturas de Marruecos durante el período 2003 - 2013.

Métodos: Se utilizó exclusivamente la información publicada disponible, a saber, los datos de HLIR del período 2003 - 2013 en el país, los cuales se obtuvieron de los archivos de libre acceso del Ministerio de Salud. Se examinaron las tendencias de largo plazo usando los coeficientes de correlación por rangos de Kendall. Se realizó

además un análisis exploratorio de los datos espaciales, a fin de examinar la auto-correlación espacial (índice de Moran global e indicador local de asociación espacial [LISA]), y la difusión espacial a nivel de las provincias/prefecturas. Se evaluó la influencia que ejercen en la HLIR diversas covariables (índice de pobreza, índice de vulnerabilidad, densidad de población y urbanización) por medio de regresión espacial (estimación de mínimos cuadrados ordinarios).

Resultados: A nivel del país, no se observó variación en el largo plazo. Según el modelo de regresión de Poisson, la tasa de incidencia anual de leishmaniasis cutánea (LC) fue de 13 por cada 100.000 habitantes (95% CI = 12,9 – 13,1), y la de leishmaniasis visceral (LV), de 0,4 por cada 100.000 habitantes (95 % CI = 0,4 – 0,5). Los datos disponibles sobre HLIR se referían a una combinación de casos de LC y LV. No obstante, dado que los de LC superaban ampliamente a los de LV, puede considerarse la HLIR como tasa de incidencia de LC. A nivel provincial, mientras que se observó un incremento de la incidencia en el largo plazo en las provincias de Al Hoceima (p = 0,008), Taounate (p = 0,04), Larache (p = 0,002), Tétouan (p = 0,0003), Khenifra (p = 0,008), Meknes (p = 0,03) y El Kelaa (p = 0,0007), solo se constató una disminución de la incidencia en el largo plazo en la de Chichaoua (p = 0,006). Si bien hubo evidencia de aumento o descenso de la incidencia en estas provincias, en ninguna se constató concentración. Por un lado, se detectó una importante concentración de incidencia de leishmaniasis en la región nororiental de Marruecos, y por otro, zonas de baja incidencia en algunas zonas del noroeste y el sur del país. No se detectó ningún patrón espacial específico en las demás regiones. Durante el período 2005 - 2013, se observó una importante concentración de la incidencia de leishmaniasis, siendo la provincia de Errachidia en todo momento la más afectada. El índice de Moran global aumentó de 0,2844 (p = 0,006) en 2005 a 0,5886 (p = 0,001) en 2011, y descendió a 0,2491 (p = 0,004) en 2013. Se observó que la pobreza fue la única variable que influyó en la tasa de incidencia de leishmaniasis humana (p = 0,0003), contribuyendo solo en un 23% (R^2 ajustado = 0,226).

Conclusión: Se han identificado localidades que presentan, ya sea un incremento de leishmaniasis humana en el largo plazo, o una importante concentración de LH. Ello puede orientar a los responsables de la toma de decisiones en cuanto a dónde asignar recursos e instrumentar medidas de lucha contra la enfermedad. Por otra parte, se exhorta a los investigadores a que realicen estudios adicionales en esas localidades específicas.

Translated from English version into Spanish by Mónica Algazi, through

TRANSLATORS
WITHOUT BORDERS
